# Novel Bioformulations Developed from *Pseudomonas putida* BSP9 and Its Biosurfactant for Growth Promotion of *Brassica juncea* (L.)

**DOI:** 10.3390/plants9101349

**Published:** 2020-10-12

**Authors:** Isha Mishra, Tahmish Fatima, Dilfuza Egamberdieva, Naveen Kumar Arora

**Affiliations:** 1 Department of Microbiology, Babasaheb Bhimrao Ambedkar University, Vidya Vihar Raebareli Road, Lucknow 226025, India; ishamishra111@gmail.com (I.M.); tahmish.dan@gmail.com (T.F.); 2 Leibniz Centre for Agricultural Landscape Research (ZALF), 15374 Müncheberg, Germany; 3 Faculty of Biology, National University of Uzbekistan, Tashkent 100174, Uzbekistan; 4 Department of Environmental Science, School for Environmental Sciences, Babasaheb Bhimrao Ambedkar University, Vidya Vihar Raebareli Road, Lucknow 226025, India

**Keywords:** *Pseudomonas putida*, PGPR, biosurfactant, bioformulation, glycolipids, rhamnolipids, *Brassica juncea*, sustainable agriculture

## Abstract

In this study, *Pseudomonas putida* BSP9 isolated from rhizosphere of *Brassica juncea* was investigated for its plant growth promoting and biosurfactant producing activities. The isolate showed the ability to produce indole acetic acid, siderophore, phosphate solubilization activity and was an efficient producer of biosurfactant. Purification (of the biosurfactant) by thin layer chromatography (TLC) and further characterization by Fourier transform infrared spectroscopy (FTIR) revealed that biosurfactant produced by the isolate belonged to the glycolipid category, which is largely produced by *Pseudomonas* sp. In addition, liquid chromatography-mass spectroscopy (LC-MS) analysis showed the presence of a mixture of six mono-rhamnolipidic and a di-rhamnolipidic congeners, confirming it as a rhamnolipid biosurfactant. Bioformulations were developed using BSP9 and its biosurfactant to check their impact on promoting plant growth in *B. juncea*. It was noted from the study that bioformulations amended with biosurfactant (singly or in combination with BSP9) resulted in enhancement in the growth parameters of *B. juncea* as compared to untreated control. Maximum increment was achieved by plants inoculated with bioformulation that had BSP9 plus biosurfactant. The study also suggested that growth promotion was significant up to a threshold level of biosurfactant and that further increasing the concentration did not further enhance the growth parameter values of the plant. The study proves that novel bioformulations can be developed by integrating plant growth promoting rhizobacteria (PGPR) and their biosurfactant, and they can be effectively used for increasing agricultural productivity while minimizing our dependence on agrochemicals.

## 1. Introduction

Agricultural productivity is a worldwide concern and there is continuous pressure on this sector to meet the rising food demands of the ever-growing population. A report by the Food and Agricultural Organization (FAO) suggests that the world population will reach nine billion by 2050, and hence global agricultural production must increase by 70% [1]. In the race to enhance crop productivity, humans are adversely impacting the environment by applying high amounts of fertilizers and using intensive agronomic practices. Climate change coupled with natural disasters has further exacerbated the situation [2,3]. Therefore, there is an urgent need to develop sustainable farming methods that are biological in origin and ecofriendly in nature. The role of plant growth promoting rhizobacteria (PGPR) has been widely exploited in the context of sustainable agricultural productivity [4]. Among various beneficial roles that PGPR confer to their host plant including production of growth enhancers, phytohormones, nutrient assimilation, metal chelation, they are also well known for their synthesis of surface-active compounds known as biosurfactants [5,6,7]. Biosurfactants are biomolecules that are amphiphilic in nature and include a large category of glycolipids, lipopeptides phospholipids, fatty acids and polymeric macromolecules [8,9]. Members of genus *Pseudomonas* are among the front runners in this context and are reported to produce cyclic lipopeptide and rhamnolipid (a type of glycolipid) biosurfactants [10,11]. Biosurfactants are biodegradable, biocompatible, durable in a range of temperatures and pH and are non-toxic in nature [9,12]. Apart from these ecofriendly and beneficial properties, it has been reported that biosurfactants produced by PGPR take part in plant growth promotion by helping in swarming motility during root colonization (by PGPR), root differentiation, nutrient acquisition, metal chelation, conferring protection against diseases and phytopathogens and providing tolerance against abiotic stresses [13,14,15,16]. They have been used as excellent candidates for biodegradation of certain organic pollutants including polyaromatic hydrocarbons and other xenobiotic compounds, thus acting as efficient bioremediation agents [17,18]. These properties of biosurfactants and their producing microbes demonstrate their potential as an environmentally friendly solution for improving crop productivity in a sustainable manner.

*Brassica juncea* (L. Czern & Coss) or Indian mustard is an important oilseed crop, which is amphiploid species and belongs to the family Brassicaceae (Cruciferae) [19]. It is a *rabi* crop and is one of the richest sources of protein, iron (Fe), vitamin A and C and also contains calcium, potassium, riboflavin, β-carotene and phenolic antioxidants such as sinapic acid and sinapine [20,21,22]. It has a low amount of saturated fatty acids (5–7%) and a high level of unsaturated fatty acids (about 7–10% α-linolenic and 17–21% linoleic acids), and thus it is considered as a healthier option in comparison to other edible oils [23]. Apart from this, it possesses glucosinolates, isothiocyanates, allyl thiocyanates and phytosterols, which have medicinal properties and antimicrobial characteristics [24,25,26]. Globally, it is the third most important oilseed crop after soybean and palm [27]. Canada is the largest producer of mustard and China holds the second largest market share. India ranks third in terms of area and production and fifth according to yield per hectare after Germany, France, Canada and China [28]. Therefore, in order to meet the ever-growing demands of oilseed in the country, it is important to increase the production of Indian mustard. For this, the use of PGPR and their metabolites in the form of bioinoculants is the best possible substitute for their chemical counterparts. They can improve plant health, productivity and also protect plants from abiotic stresses, thus minimizing reliance on synthetic inputs. In the present study talc-based bioformulations were developed using a rhizospheric pseudomonad BSP9 and its metabolite (biosurfactant) to check their impact on the growth of *B. juncea* in field conditions.

## 2. Results and Discussion

### 2.1. Isolation and Identification of Isolate

In total, 15 bacterial isolates were obtained from rhizosphere of *B. juncea* and BSP9 was selected on the basis of its potent plant growth promoting (PGP) and biosurfactant producing properties. The morphological characteristics showed that the isolate is Gram negative, motile, rod shaped, and forms smooth and shiny colonies with convex and entire margins. Biochemical examinations revealed that the bacteria were positive for catalase, oxidase, nitrate reduction, citrate utilization, indole and gelatin hydrolysis and negative for methyl red, Voges-Proskauer and starch hydrolysis, suggesting it was *Pseudomonas* sp. [29]. The isolate was further processed for molecular identification through Nucleotide-BLAST. The 16S rRNA sequence of the isolate was found to be clustered with *Pseudomonas putida* strain ATCC 12,633 (Accession No. NR 114479), showing a similarity of 99.6%. The homology of the same was also evident from the phylogenetic tree (Supplementary Materials, Figure S1). The sequence data was submitted to GenBank with Accession No. LC489268. The strain was also submitted to the National Agriculturally Important Microbial Culture Collection, an international culture collection center approved by the International Depository Authority (IDA), with Accession number *P. putida* NAIMCC-B-02326. Studies reveal that the *P. putida* bacteria is ubiquitous and is well known for its metabolic versatility, adaptability and PGP properties. Hence, these bacteria are considered as excellent candidates for the development of bioinoculants and as soil enhancers to increase crop yields [30].

### 2.2. PGP Characterization of the Isolate

PGP characterization of isolate BSP9 showed positive results and confirmed that it is a solubilizer of phosphate as well as a producer of indole acetic acid (IAA) and siderophore. Phosphorus (P) is an essential macronutrient for plants and serves as a crucial element for the photosynthetic, metabolic and biochemical pathway development and growth of plants [31]. Despite its abundance in soil, most P is unavailable to plants due to the fixation of insoluble phosphates, and hence is a major limiting factor in agricultural systems [32]. In the present study, isolate *P. putida* BSP9 showed phosphate solubilization of 3.6 PSI (unit for phosphate solubilization index). Research suggests that pseudomonads, including *P. putida* are known to possess excellent phosphate solubilizing properties, which render them accessible to plants and reduces the load of synthetic P fertilizers [33,34]. In addition, another phosphate-solubilizing *P. putida* showed significant dissolution of phosphate as well as increased root and shoot dry weight of corn compared to the control [35]. Similarly, an auxin-producing *P. putida* MTCC 5279 (RAR) showed considerable solubilization of inorganic P and also promoted growth of *Arabidopsis thaliana* through modulation of biochemical and physiological processes in plants under P deprived-salinity stress conditions [36]. IAA production by plant-associated bacteria is also widespread and it is one of the most important traits of PGPR. IAA is directly involved in the growth and proliferation of plant roots and enhances their ability to acquire water and nutrition from soil [37,38]. Tests for IAA production revealed that BSP9 produced 31.90 µg/mL of IAA. In the same way, *P. putida* strain AF137 isolated from rice cultivars in Afghanistan soils produced 92.30 μg mL^−1^ and its inoculation enhanced root and shoot dry weights of rice [39]. Similarly, a rhizobacterial strain of *P. putida* FA-56 was assessed for in vitro IAA production and its inoculation significantly increased the growth parameters of tomato plants [40]. The production of iron chelating compounds known as siderophores by rhizospheric bacteria is another important PGP trait [41]. Iron sequestration helps plants in the formation of chlorophyll, is involved in electron transfer, helps in the synthesis of DNA and RNA as well as their repair, and also acts as an antagonistic agent against phytopathogens [42,43]. Siderophore production by BSP9 was checked and it was found to produce 44.74% psu (percent siderophore unit). *P. putida* KT2440 has been reported to be beneficial to plants as it produces pyoverdine-siderophore and is involved in a homeostasis mechanism that mitigates oxidative stress [44]. Iron chelating *P. putida* MPJ6 produced 85.3% siderophore under iron-deficient conditions and its biofortification increased iron content and other vegetative parameters in mungbean [45]. The above results suggest that BSP9 is an efficient plant growth promoter with multiple PGP properties, which correlates well with earlier findings, thus indicating its potential to be used as a bioinoculant.

### 2.3. Production, Extraction and Purification of Biosurfactant by Isolate BSP9

The production of biosurfactant by isolate BSP9 was checked. The test showed positive results for hemolysis activity and produced clear zones around the streaked area, which suggested the probability of biosurfactant production. The presence of biosurfactant was further confirmed by the blue agar plate (BAP) test, which was found to be positive due to the presence of dark blue halo zones around spot inoculation (Supplementary Materials, Figure S2). It was also found that BSP9 showed 59.95% (E_24_) activity, thus confirming the production of biosurfactant. Further, the extraction process was carried out and after filtrating and evaporating the organic layer, a dark brown-colored viscous residue was left over (crude biosurfactant) and was weighed to be 2.5 g/L. For purification, thin layer chromatography (TLC) was done and the analysis of the two extracted major spots (retention factor Rf = 0.33 and Rf = 0.78) (Supplementary Materials, Figure S3) showed mobility similar to that of glycolipids [46,47]. The spot nearest to the point of origin (Rf = 0.33) represented di-rhamnolipid while the spot further away from the point of origin (Rf = 0.78) corresponded to mono-rhamnolipid [48,49]. Biosurfactants have gained special attention in recent times owing to their wide range of applications, operational flexibility, low toxicity and ecofriendly nature [50]. Jarvis and Johnson [51] first isolated and described the presence of rhamnolipids biosurfactant in *Pseudomonas aeruginosa*. However, due to the pathogenic nature of this bacterium, there were safety issues related to its production and usage that need to be addressed and evaluated before its utilization in any field [52,53]. Recently, interest in these naturally-derived products has increased significantly and the present range of microbial surfactants needs to be expanded by identifying non-pathogenic sources for enhanced production [54]. Thus, investigating the role of biosurfactant-producing isolate BSP9 in the current study is important for its future utilization in various areas. Although *P. putida* has been reported to produce different types of biosurfactants, mostly rhamnolipids have been reported [55,56,57]. However, the present study reports a strain of *P. putida* BSP9 with PGP characteristics including phosphate solubilization, production of IAA and siderophore along with biosurfactant. Because of these novel characteristics, the strain has been submitted in the international culture collection center as well.

### 2.4. Structural Characterization of the Biosurfactant

**Figure 1:**
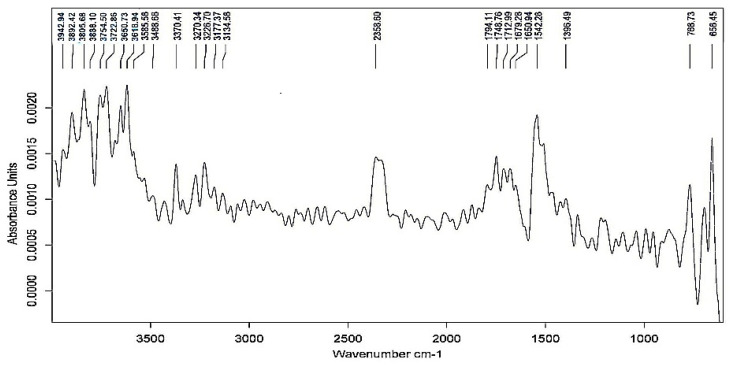
FTIR spectrum of the functional groups present in purified biosurfactant produced from BSP9.

The Fourier transform infrared spectroscopy (FTIR) analysis of the biosurfactant produced from BSP9 clearly reveals the presence of functional groups that typically belong to glycolipids. The spectrum (Figure 1) was consistent with the structure of glycolipids already reported in the literature [58,59,60]. The IR spectrum shows absorption bands ranging between 3650–3134 cm^−1^, which indicate the presence of free -OH group arising as a result of H bonding of polysaccharides and/or –OH stretching of carboxylic acid. Two bands at wavenumber 1748 cm^−1^ and 1712 cm^−1^ denote –C=O stretching arising from the ester groups of lipids and fatty acids (-C=O in COOH), and bands at wavenumber 1396 cm^−1^ signify C-H and OH deformation vibrations of carbohydrates that may be arising due to the presence of rhamnose units in the biosurfactants [61]. A small peak at wavenumber 2358 cm^−1^ is likely due to the presence of phosphines (P-H_3_) in the biosurfactant [59]. The range of spectra below 1000 cm^−1^ represents C-H, C-O and CH_3_ vibrations [62]. Glycolipid biosurfactants possess carbohydrate moieties linked to fatty acid (as evident from the FTIR data) and are largely produced by *Pseudomonas* sp. [63]. Rhamnolipids, trehalolipids, mannosylerythritol lipids and cellobiose lipids belong to the category of glycolipids [64]. Glycolipids are among the most promising biosurfactants and have shown high productivity and extreme biochemical versatility, and hence they are being used in a wide range of industries including agriculture [64,65].

**Figure 2:**
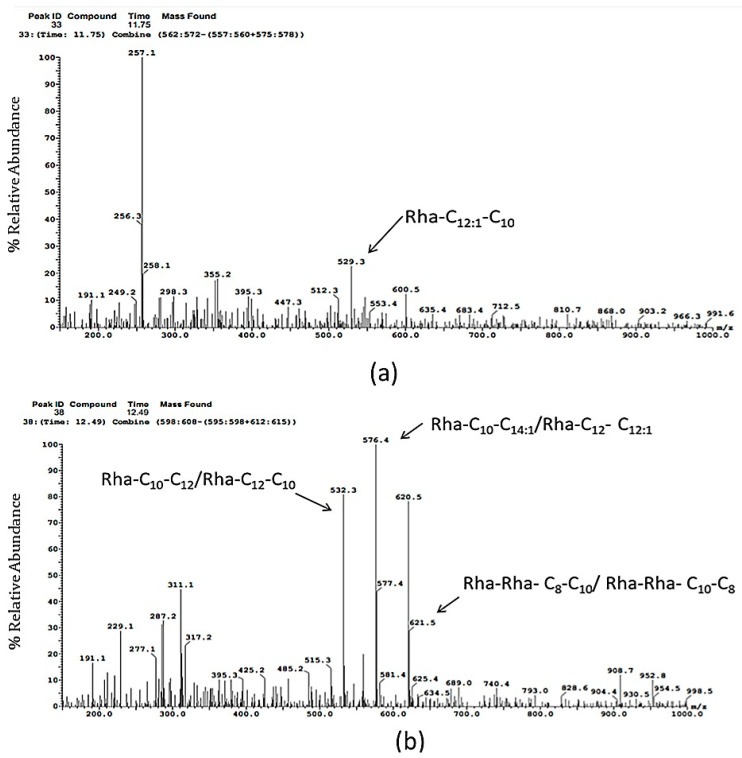

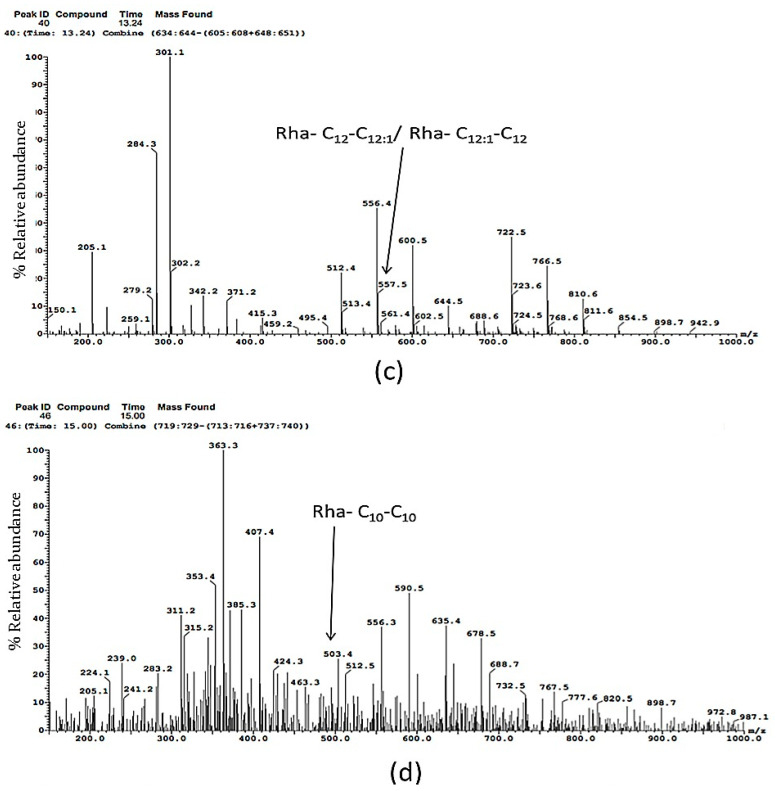
LC-MS analysis of the biosurfactant produced from BSP9 showing specific rhamnose congeners (**a**) peak at *m*/*z* 529.3 marked by arrow corresponds to Rha-C_12:1_-C_10_ LC-MS (**b**) peaks at *m*/*z* 532.3, 576.4 and 621.5 marked by arrows correspond to Rha-C_10_-C_12_/Rha-C_12_-C_10_, Rha-C_10_-C_14:1_/Rha-C_12_-C_12:1_ and Rha-Rha-C_8_-C_10_ or Rha-Rha-C_10_-C_8_ respectively (**c**) peak at *m*/*z* 557.8 corresponds to Rha- C_12_-C_12:1_/Rha- C_12:1_-C_12_ (**d**) peak at *m*/*z* 503.4 corresponds to Rha-C_10_-C_10_.

The characteristic rhamnolipid moieties in the extracted biosurfactant were identified using LC-MS data (Figure 2). It was evident from the analysis that rhamnose analogues detected at *m*/*z* 529.3 with a retention time of 11.75 min correspond to Rha-C_12:1_-C_10_ (Figure 2a). The same mass range was obtained by LC-MS analysis of rhamnolipids produced by *P. aeruginosa* strain ATCC 9027 [66]. Likewise, ionization at *m*/*z* 532.3 and 576.4 with a retention time of 12.49 min correspond to Rha-C_10_-C_12_/Rha-C_12_-C_10_ and Rha-C_10_-C_14:1_/Rha-C_12_-C_12:1_, respectively (Figure 2b). In their study, Bajpayi et al. [67] reported similar rhamnose congeners produced by *Pseudomonas protegens* strain BNJ-SS-45 at a similar mass range. Molecular ions at *m*/*z* 557.5 (retention time = 13.24 min) (Figure 2c) and 503.4 (retention time = 15.00 min) (Figure 2d) showed the presence of Rha-C_12_-C_12:1_/Rha-C_12:1_-C_12_ and Rha-C_10_-C_10_, respectively. The LC-MS study of rhamnolipids produced by *Pseudomonas gessardii* showed similar rhamnolipidic moieties with the same mass range [68]. LC-MS analysis in the present study revealed the abundance of six compositionally different homologues of mono-rhamnolipid congeners. However, a di-rhamnose congener, i.e., Rha-Rha-C_8_-C_10_ or Rha-Rha-C_10_-C_8_ was also obtained at *m*/*z* 621.5 with a retention time of 12.49 min (Figure 2b), which was also evident in the study by Sabturani et al. [69]. Rhamnolipids are predominantly produced by *Pseudomonas* sp. containing mono, di-rhamanolipidic congeners or a mixture of both, and there are more than 100 known homologues of rhamnolipids produced by *Pseudomonas* sp. that have been reported until now [70]. Earlier findings suggested that mono-rhamnolipidic moieties act as a wetting agent for bacteria while on the other hand, a di-rhamnolipidic congener has been found to work as a self-produced chemotactic attractant by some *Pseudomonas* sp. [53,71].

### 2.5. Field Trial

To check the efficacy of rhamnolipids and BSP9 as a bioinoculant, talc-based bioformulations were developed using nine different treatments, which included metabolite (purified biosurfactant), only cells (BSP9), a combination of both (biosurfactant and BSP9 cells) and an untreated control. *B. juncea,* being an important oilseed crop (in this region) was selected for field trial and its seeds were treated with prepared bioformulations. The soil at the field site had a pH = 7.5, EC 1.5 dS/m, moderate organic matter 5.4 g/Kg, available nitrogen (N) 148.3 kg/ha, P 18.2 kg/ha, and available potassium (K) 163 kg/ha. The field trial revealed significant enhancement in the germination rate (82.3%), root length (100.8%), shoot length (81%), fresh weight (66.3%), dry weight (79%), number of pods (69.3%), oil content (28.6%) and phytochemical contents, i.e., chlorophyll (94.2%) and flavonoid (112.1%) in plants treated with 2% biosurfactant plus BSP9 compared to the control (Table 1). The results of 5% biosurfactant plus BSP9 were very similar, hence they are not shown. Also, the production cost of 2% biosurfactant for preparation of bioformulations would be more economical than 5%. The results for the growth parameters after treatment with only BSP9 cells also showed significant improvement but were lower compared to the biosurfactant and BSP9 together. Interestingly, when only biosurfactant was used in the bioformulation, an increasing trend in growth parameters was observed with an increase in its concentration (0.1%, 1% and 2%) and the values dipped after 2%, although only slightly.

Multiple PGP traits of BSP9 including production of IAA, siderophore and solubilization of phosphate could be the main reason for the growth promotion of Indian mustard. However, an additional property of the isolate, i.e., the potential to produce biosurfactant conferred added benefits to the plants. Research studies have shown that inoculation of rhamnolipid-producing microbes can induce non-specific immunity to plants against biotic and abiotic factors [72]. Studies have also revealed that rhamnolipid-producing PGPR synthesize quorum-sensing molecules such as acyl homoserine lactone (AHL) [73]. These signaling molecules have been reported to increase the interaction between PGPR and the plant root, improving their colonization, and thus resulting in yield increment [13]. In addition, external amendment of rhamnolipid biosurfactants in bioformulation may also have helped bacterial cells by increasing their nutrient chelating ability to form complexes with important metal ions and micronutrients in soil [74]. Also, their potent penetrating action, gelling, wetting and amphiphilic properties make these biosurfactants excellent dispersing agents [75], which could confer great advantage to bioformulations by helping the colonization of plant roots (by PGPR) and making phytohormones and siderophores available to the plant. Earlier reports show that biosurfactants have been used as carriers and penetrants in chemical fertilizers and pesticide products to increase their translocation at a determined rate on treated plants [76,77]. The substantial increase in plant growth parameters of *B. juncea* treated with BSP9 plus biosurfactant could be attributed to the above factors, which may have played a determining role in enhancing crop productivity. Biosurfactants are non-toxic and easily degradable [9], hence only BS-based bioinoculants may get degraded in the complex rhizosphere ecosystem, which has a huge diversity of microorganisms. Thus, the use of PGPR with the ability to produce BS provides a possible solution because it continuously release these important biomolecules in the soil. The present study also showed that the use of a combination of PGPR and BS was most effective. It will also be important to determine the exact mechanisms of plant growth promotion by BS, which are not very clear at present. Hence, further research is required to define their role in plant–microbe and microbe–microbe interactions in the rhizosphere. For commercial applications it will be essential to bring down the cost of production of BS by optimizing the conditions and the use of cheap carbon sources.

**Figure 3:**
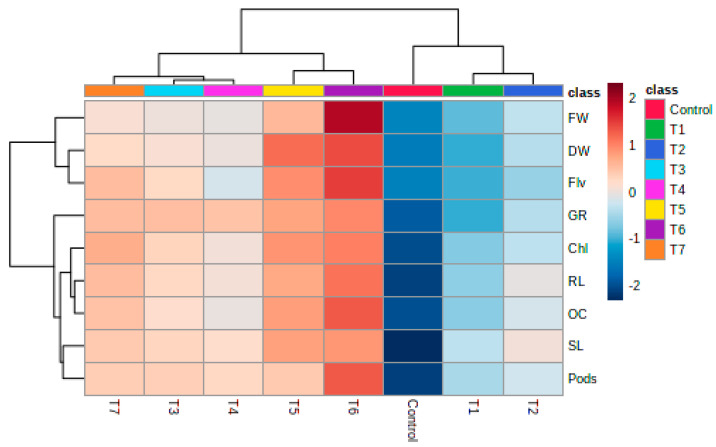
Heatmap generated to determine distance and clustering and showing the effect of biosurfactant and BSP9 on various growth parameters of Indian mustard in a field study (distance measure using Euclidean and clustering algorithm using Ward’s method). T1 to T7 and control are different treatments (T1-0.1% biosurfactant (BS), T2-1.0% B.S, T3-2.0% BS, T4-5.0% BS, T5-1% BS + BSP9, T6-2% BS + BSP9, T7-only BSP9 cells and Control = untreated control). FW-fresh weight, DW-dry weight, Flv-flavonoid content, GR-germination rate, Chl-chlorophyll content, RL-root length, OC-oil content, SL-shoot length, Pods-number of pods.

Figure 3 (heatmap) highlights the hierarchical clustering of the treatments and the associated effect on the growth parameters of mustard plants. As shown in the dendrogram, there are two major classifications, one including the untreated control, 0.1% and 1% biosurfactant (BS)-treated plants with lower growth parameters. The other major classification included higher concentrations of BS (2% and 5%), bacteria only and metabolite and bacterial combination treatments. Interestingly, the treatments further showed two types of variations, first the combination (1% and 2% BS with BSP9) applied to plants, which had the highest impact on all growth parameters and second, bacteria alone and higher concentrations of metabolite (2% and 5%) application, which had comparatively less impact. The most interesting conclusion from the clustering analysis is that although bacteria and metabolite synergistically are outstanding growth promoters, 2% and 5% BS (treatment alone) has almost the same impact as BSP9. Therefore, it can be suggested that metabolites like biosurfactant are capable of directly increasing the plant growth and yield in addition to their remediation and biocontrol activities. Analysis of the clustering algorithm, also suggests that lower concentrations of BS (0.1% and 1% BS) are incapable of stimulating significant plant growth as compared to other treatments. The study also highlights that there is a threshold of metabolite (biosurfactant) concentration required to enhance productivity and yield of plants due to causes unknown, which could be the subject of future study. Chaprão et al. [78] explained the effect of concentration in a study that reported that the application of more than 40 mg/mL biosurfactant to soil reduced diesel biodegradation efficiency. This was confirmed by Fenibo et al. [12], who concluded that if the optimum concentration of biosurfactant is exceeded, the microbial biomass decreases and the degradation process is also negatively affected. However, there are no previous reports about optimized biosurfactant concentration for direct plant growth promotion under field conditions, hence, the present work is a novel report that extends the knowledge about the biopolymer.

Owing to its multiple PGP properties including phosphate solubilization and the production of IAA, siderophore and biosurfactant-producing ability and non-pathogenic nature, isolate BSP9 can be considered as a suitable candidate for the preparation of biosurfactant-based bioformulations. Agriculture is heavily dependent on chemical-based fertilizers and pesticides, and hence the development of novel and effective bioformulations to enhance crop productivity has become very important. PGPR-based formulations supplemented with biosurfactant could be an organic alternative to chemical inputs in farming practices with the added advantages of better performance and colonization of the bacterium in plant roots. Application and commercialization of such bioformulations can be exploited to promote plant growth and to serve as a future approach to increase crop production.

## 3. Materials and Methods

### 3.1. Isolation and Identification of the Isolates

Bacteria were isolated from rhizosphere of *B. juncea* from the Lucknow region (26.8467° N, 80.9462° E) and were identified based on morphological and biochemical tests according to Bergey’s Manual of Systemic Bacteriology [29]. Out of all the isolates, BSP9 was selected on the basis of its efficient plant growth promoting properties (PGP). Further, molecular identification of the isolate BSP9 was performed, for which 16S rRNA gene was amplified with universal primers (27F 5′-AGAGTTTGATCMTGGCTCAG-3′ and 1492R 5′-GGTTACCTTGTTACGACTT-3′) using polymerase chain reaction (PCR) according to Srinivasan et al. [79]. Nucleotide-BLAST was used to identify the sequence homology. The sequences identified after homology searching were subjected to clustering and development of the phylogenetic tree. Isolate BSP9 was submitted to NAIMCC, Uttar Pradesh, India.

### 3.2. Plant Growth Promoting Characters of the Isolate BSP9

To check the plant growth promoting (PGP) potential of the isolate BSP9 phosphate solubilization, production of IAA and siderophore activity were determined. Evaluation of phosphate solubilizing activity was carried out using Pikovskaya’s medium [80] and the solubilization index (SI) was calculated by measuring the diameter of clear zone. The quantitative estimation of IAA production was obtained by colorimetric analysis [81]. IAA production was detected in culture filtrate using minimal broth supplemented with L-tryptophan (2 mg/mL). Siderophore production by the isolate was checked on Chrome-Azurol S (CAS) agar plates [82] and CAS broth using microtiter plate method as described by Arora and Verma [83].

### 3.3. Production, Extraction and Purification of Biosurfactant by Isolate BSP9

The production of biosurfactant by BSP9 was checked by performing screening tests, which were as follows: hemolytic assay [84], oil spreading test [85], and BAP assay [86]. The emulsification index was evaluated by the formula given below [87]:

Emulsification index (E_24_) = height of emulsion formed/total height × 100 (1)

For the extraction process, the log phase culture (24 h) of BSP9 (with OD 610 = 0.1 CFU 10^8^/mL) was inoculated in Bushnell Haas broth (Himedia, Mumbai, India) with the following composition (g/L): magnesium sulfate (MgSO_4_) 0.2, calcium chloride (CaCl_2_) 0.02, monopotassium phosphate (KH_2_PO_4_) 1.00, dipotassium phosphate (K_2_HPO_4_) 1.00, ammonium nitrate (NH_4_NO_3_) 1.00, ferric chloride (FeCl_3_) 0.05, supplemented with 1% glycerol as carbon source and pH was adjusted to 7.0. After 72 h of incubation, the broth was centrifuged at 10,000 rpm for 20 min. Supernatant was collected in a fresh tube, acidified to pH 2.0 using 6N HCl and incubated overnight at 4 °C. Further, biosurfactant was extracted using a mixture of chloroform:methanol in a ratio of 2:1 (*v*/*v*) in a separating funnel and was then centrifuged at 10,000 rpm for 15 min. Solvent extract was filtrated using 0.45 mm Millipore membrane (Merck, Mumbai, India) and evaporated to dryness in an oven [88]. Finally, a semi-purified dark brown viscous residue was collected.

Thereafter, for purification, the crude precipitate was run on a TLC plate. The solvent system used was chloroform:methanol:water in the ratio 65:15:2 (*v*/*v*/*v*). The spots were produced by spraying anthrone-sulfuric acid reagent and heated at 110 °C for 20 min [47]. The spots were scraped, re-extracted in chloroform:methanol and evaporated to dryness [89].

### 3.4. Structural Characterization of the Biosurfactant

For identification of the functional groups in the purified biosurfactant, FTIR analysis was done using a Bruker Avance 600 instrument. For this, 1 mg of freeze dried biosurfactant was mixed with 100 mg of KBr and a thin translucent pellet was placed to record the IR in the range 400 cm^−1^ and 4000 cm^−1^ [90].

For further detection of the structural composition, liquid chromatography–mass spectrometry (LC-MS) was performed according to Haba et al. [91] with slight modifications using Waters UPLC-TQD Mass Spectrometer (Waters, MA, USA). C18 column (250 × 4.6 mm, 5 μm pore size) was used with an acetonitrile-water system at a flow rate of 0.3 mL/min. Rhamnolipid congeners were identified based on mass spectrum, which ranged from *m*/*z* 150–1000.

### 3.5. Field Study

The field trial was conducted during *rabi* season for two consecutive years, i.e., 2017–2018 and 2018–2019 at an agricultural farm near Lucknow, Uttar Pradesh (U.P.) located at 26.8467° N, 80.9462° E. In two years of field trial, the maximum and minimum temperature ranged between 20–40 °C and 7–25.9 °C, respectively, during crop growing season. The chemical properties of the soil including pH, electrical conductivity (EC), organic carbon, available N, P, and K were checked according to the standard protocols [92,93]. The field experiment was done in triplicate with completely randomized block design (CRD) (size of each block = 2.43 m × 2.43 m) taking *B. juncea* as the test crop. The field was irrigated thrice by ground water between sowing and harvesting. Varuna-T56 variety of *B. juncea* was selected as it is the most common seed type used by farmers of the region. It is a short duration cultivar that suits the uneven climatic and edaphic factors of the region [94]. Before sowing, the seeds were washed and surface sterilized with 3% hydrogen peroxide followed by washing with sterilized distilled water 4 to 5 times. Talc-based bioformulations were then developed according to the method of Nandakumar et al. [95]. For this, 1 kg of talc powder was used and pH was adjusted to neutral by adding CaCO_3_ at the rate of 15 g/kg talc. Carboxymethyl cellulose (CMC) (1 g) was added into this mixture in order to increase the adhesion properties of the bioformulation and the mixture was sterilized by autoclaving. Thereafter, bacterial inoculum suspension was prepared by growing BSP9 in nutrient broth (NB) medium (Hi-Media) and incubation in a rotary shaker at 150 rpm and 27 ± 2 °C. After growth, 400 mL of log phase cell suspension (OD 610 adjusted to ~1) was added to the sterilized carrier-cellulose mixture. Bacterial population density was measured by taking 1 g of bioformulation in 10 mL of distilled water and serially diluting it to 10^–6^ and 10^–7^ [96]. Bacterial density in the bioformulation mixture was recorded to be ~10^8^ CFU/g. Surface sterilized seeds were dipped in the suspension and left overnight for proper coating. The bacterial population density on seeds was found to be ~10^7^ CFU/seed. After this, biosurfactant was amended in various concentrations, i.e., 0.1, 1.0, 2.0 and 5.0% weight/volume. The combination of metabolite and BSP9 was prepared by adding biosurfactant at the rate of 10 mg *w*/*v* (for 1% biosurfactant + BSP9), 20 mg *w*/*v* (for 2% biosurfactant + BSP9) and 50 mg *w*/*v* (for 5% biosurfactant + BSP9) to 1000 mL of talc suspension containing BSP9 cells. The cell density of the bioformulation containing bacterial cells and metabolite was measured and found to be ~10^7^ CFU/seed. After overnight treatment, seeds were dried for 2–3 h and 300 were sown in each block (in triplicate) according to treatments: 0.1% biosurfactant, 1.0% biosurfactant, 2.0% biosurfactant, 5.0% biosurfactant, 1.0% biosurfactant + BSP9, 2.0% biosurfactant + BSP9, 5.0% biosurfactant + BSP9, and untreated control. Plants were uprooted 120 days after sowing (DAS). Ten plants from each treatment were used for analysis of the growth parameters including root and shoot length, fresh and dry weight, number of pods and oil content. For phytochemical analysis, ten plants from each treatment were uprooted 90 DAS and their fresh leaves were homogenized to check the chlorophyll [97] and flavonoid contents [98].

All the data for the plant growth parameters were statistically analyzed using one-way analysis of variance (ANOVA) and Duncan’s Multiple Range Test (DMRT) at 5% level to compare differences between treatment means. Data was analyzed by the software statistical package for the social sciences (SPSS) (2016) for Windows. A heatmap was drawn using R-package (version 3.6.2) to visualize the effect of various treatments on plant growth parameters through clustering analysis.

## 4. Conclusions

Based on this study, it can be concluded that the use of plant growth promoting strain *P. putida* BSP9 and the rhamnolipid biosurfactant it produces is a novel technique for enhancing the productivity of *B. juncea.* It was observed that the combination of *P. putida* BSP9 and rhamnolipid BS showed the maximum enhancement in the growth parameters of *B. juncea*, including root and shoot length, total fresh and dry weight, number of pods, total oil content, total chlorophyll and flavonoid content. The study also suggested that optimization of biosurfactant is important to develop bioinoculants to achieve maximum plant growth promotion. After a threshold/optimum amount of biosurfactant (i.e., 2% BS in the bioinoculant) the values of plant growth parameters started to decline. The stimulatory effects of these biosurfactants can be utilized to enhance the production of other crops to achieve the targets of food security. The application of these microbial-metabolite-based products is an environmentally sustainable approach that improves crop productivity and could minimize our reliance on agro-chemicals. However, further research is required to determine the exact mechanisms of biosurfactants and the role these important metabolites play in plant-microbe interactions. It would also be interesting to examine the potential of an efficient BS-producing PGPR strain such as *P. putida* BSP9 in rhizoremediation.

## Figures and Tables

**Table 1:** Effects of inoculation of different treatments on growth parameters of *B. jumcea.*

Treatment	% Germination	Root Length (cm)	Shoot Length (cm)	Total Fresh Weight (gm)	Total Dry Weight (gm)	Number of Pods	Total Oil Content (%) *	Total Chlorophyll Content (mg/g)	Total Flavonoid Content (mg/g)
**C**	50.1 ± 0.5 ^a^	15 ± 0.4 ^a^	119.3 ± 0.5 ^a^	290.8 ± 1.4 ^a^	114 ± 1.02 ^a^	284.2 ± 8.7 ^a^	35.0%	16.6 ± 0.1 ^a^	6.4 ± 0.2 ^a^
**T1**	62.3 ± 0.9 ^b^	21.9 ± 0.1 ^b^	179.7 ± 1.3 ^b^	323.4 ± 4.2 ^b^	129.2 ± 1.9 ^b^	379.8 ± 11.3 ^b^	39.0%	23.4 ± 0.16 ^b^	7.5 ± 0.2 ^b^
**T2**	71.5 ± 0.6 ^c^	24.6 ± 0.3 ^c^	191.3 ± 1.4 ^c^	354.5 ± 2.9 ^c^	149.2 ± 0.8 ^c^	394 ± 4.7 ^b^	40.5%	25.2 ± 0.1 ^c^	8.5 ± 0.1 ^c^
**T3**	84.9 ± 0.6 ^d^	26.1 ± 0.5 ^de^	198.8 ± 0.9 ^e^	374.2 ± 2.1 ^d^	164.5 ± 1.1 ^d^	426.8 ± 1.2 ^c^	41.5%	28.5 ± 0.2 ^e^	10.5 ± 0.2 ^e^
**T4**	83.9 ± 0.9 ^d^	25.2 ± 0.4 ^cd^	195.4 ± 0.5 ^d^	371.2 ± 1.9 ^d^	161 ± 1.4 ^d^	421.6 ± 2.8 ^c^	41.0%	27.2 ± 0.2 ^d^	9.5 ± 0.2 ^d^
**T5**	88.2 ± 0.7 ^e^	28.1 ± 0.1 ^f^	213.8 ± 1.1 ^g^	405.5 ± 1.7 ^e^	197.0 ± 1.2 ^e^	429.4 ± 6.6 ^c^	43.5%	31.4 ± 0.2 ^f^	12.2 ± 0.2 ^g^
**T6**	91.3 ± 0.2 ^f^	30.1 ± 0.1 ^g^	216 ± 1.8 ^g^	483.4 ± 5.3 ^f^	204 ± 4.6 ^e^	481.2 ± 2.1 ^d^	45.0%	32.3 ± 0.1 ^h^	13.5 ± 0.1 ^h^
**T7**	85.2 ± 1.1 ^d^	27.4 ± 0.5 ^ef^	202.8 ± 0.7 ^f^	379 ± 3.2 ^d^	168.6 ± 1.3 ^d^	427 ± 4.2 ^c^	42.5%	30.4 ± 0.1 ^g^	11.2 ± 0.1 ^f^

Results expressed as mean ± S.D (*n* = 10 of two years data (2017–18 and 2018–19)). Same superscript letters denote no significance difference (*p* < 0.05) determined by Duncan’s multiple range test (DMRT). T1-0.1% biosurfactant (B.S), T2-1.0% BS, T3-2.0% BS, T4-5.0% BS, T5-1% BS + BSP9, T6-2% BS + BSP9, T7-only BSP9 cells and C = untreated control. * Collective mean values of oil content have been shown.

## Supplementary Materials

The following are available online at https://www.mdpi.com/2223-7747/9/10/1349/s1: Figure S1: Phylogenetic tree of the isolate BSP9; Figure S2: Isolate BSP9 showing positive BAP test due to the presence of blue halo around culture well; Figure S3: showing TLC analysis of the crude biosurfactant produced by BSP9.
