# Supplementary material for: Novel Bioformulations Developed from Pseudomonas putida BSP9 and Its Biosurfactant for Growth Promotion of Brassica juncea (L.)
Source: Plants (Basel). 2020 Oct 12;9(10):1349. doi: 10.3390/plants9101349 (PMC7601481; doi:10.3390/plants9101349)
Supplement: Supplementary file 1 [file plants-09-01349-s001.pdf]

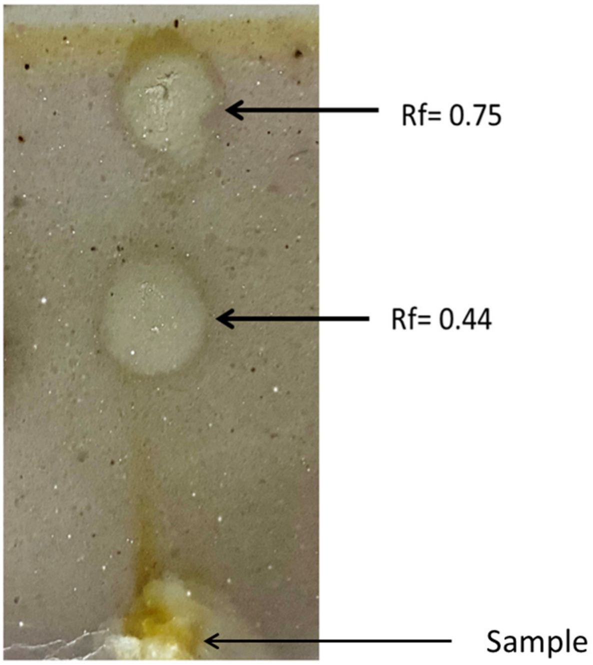

Supplementary Figure S2. showing TLC analysis of the crude biosurfactant produced by BSP9. Extracted biosurfactant show two spots with Rf= 0.44 and 0.75 confirming the presence of glycolipids

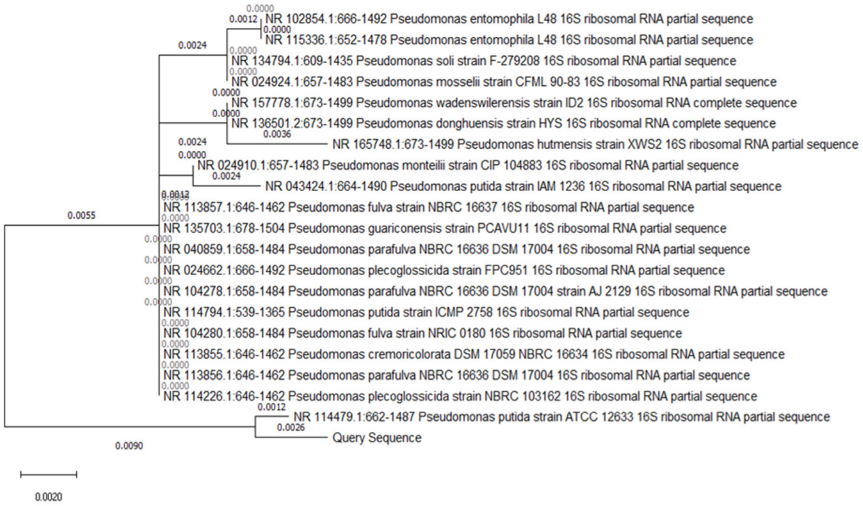

Figure S1. showing phylogenetic tree of the isolate BSP9
